# Provision of active ventilation via solar-powered fans to outdoor hutches during summer improves immune responses of dairy heifers

**DOI:** 10.3168/jdsc.2025-0962

**Published:** 2026-03-06

**Authors:** E. Tabor, G.A. Larsen, J. Laporta

**Affiliations:** Department of Animal and Dairy Sciences, University of Wisconsin–Madison, WI 53706

## Abstract

Despite the preweaning period being critical for immune system development, heat abatement for calves in summer is often overlooked during this early-life period. Herein, we evaluated selected markers of innate and adaptive immunity development in outdoor hutch-housed Holstein dairy heifers with 2 types of ventilation during a Midwestern summer. Heifer calves were housed in outdoor hutches with passive (PASS; 0.07 m/s natural air speed, n = 16) or active ventilation (ACT; 1.1 m/s air speed via solar-powered fans, n = 16) from birth to 28 d of life. At d 28, fans were turned off, and all heifers were naturally ventilated. Heifers were weaned at 49 d and monitored until 56 d of age. Blood samples were collected at various stages of the preweaning period (d 1–56) to assess IgG, glucose, blood hematology, gene expression, and functional assays. At birth, circulating IgG, glucose, and hematology parameters were not different between groups. Relative to heifers exposed to PASS, ACT heifers had reduced white blood cell, monocyte, neutrophil, and lymphocyte counts, particularly the first 28 d. The ACT heifers had a greater percentage of neutrophils with phagocytic activity on d 28 and 42 of life, and tended to have neutrophils with greater percentage of oxidative burst on d 42. The mRNA expression of haptoglobin, IL-6, IL-8, IFN-γ, and NFKβ1 was downregulated in peripheral blood leukocytes of PASS heifers, relative to ACT. Providing active ventilation to heifers during the first 4 wk of life in summer enhances neutrophil phagocytic capacity, reduces circulating neutrophil and lymphocyte counts, and downregulates the expression of key pro-inflammatory cytokine and interleukin genes in peripheral leukocytes.

## Highlights

• Active ventilation reduced white blood cells, neutrophils, monocytes, and lymphocytes.
• Ventilated calves exhibited greater neutrophil phagocytic and oxidative activity.
• Expression of key pro-inflammatory genes was downregulated.
• Early-life cooling modulated immune status and inflammatory markers.

## Graphical Abstract

**Figure:**
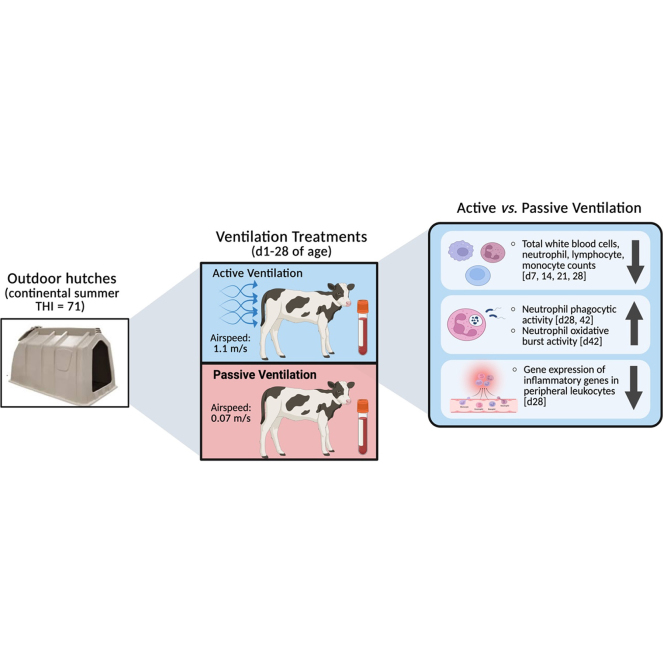
**Summary:**In this study, we evaluated the functional, cellular, and molecular responses of outdoor hutch-housed dairy heifers with or without access to active ventilation during a continental summer, testing whether early-life cooling alters immune and inflammatory responses under these environmental conditions. THI = temperaturehumidity index.

Dairy calves are born immunologically naïve, relying heavily on passive immunity from colostrum for early protection. The bovine immune system develops progressively through the first months of life, reaching full maturity between 3 and 6 mo (Cangiano et al., 2024). Calves acquire passive immunity through the transfer of antibodies from maternal colostrum, which depends on both the IgG content of colostrum, and the calf's ability to absorb IgG. Whereas colostrum-acquired IgG levels gradually decline the first month of life, active immunity to pathogens increases (Chase et al., 2008). This delicate balance between acquired and active immunity is highly sensitive to environmental stressors—particularly heat stress—which can impair IgG absorption, weaken immune maturation, and increase disease susceptibility (reviewed by Dahl et al., 2020a).

While prenatal effects of heat stress are well established, only a few studies have examined how postnatal (preweaning) heat stress influences immune function in dairy calves, and although calves remain highly developmentally plastic, exposure to elevated temperatures during this critical window may disrupt immune maturation and long-term health. Calves exposed to constant heat conditions (35°C temperature, environmentally controlled chambers) for 3 to 14 d of life had higher rates of mortality, 27% less circulating IgG, and reduced peripheral blood lymphocyte (**LYM**) counts compared with their thermoneutral counterparts (Kelley et al., 1982). Continuous exposure to pre- and postnatal environmental heat stress (i.e., last 6 wk of gestation and 8 wk postnatal, temperature-humidity index >68) led to calves having lower red blood cell (**RBC**) counts and decreased blood leukocyte mRNA expression of heat shock protein 70 and several immune-related genes relative to cooled counterparts (Marrero et al., 2021).

Although heat abatement strategies have been proven effective in maintaining thermoneutrality and immunocompetence of mature lactating and dry (late-gestation) cows and their offspring, preweaning calves are often not considered a priority for heat abatement by farmers. This may be due to the perception that preweaning calves are less susceptible to heat stress as a result of their larger surface-to-mass ratio, underdeveloped rumen, and lower metabolic heat production compared with mature cows. However, emerging evidence suggests that calves are negatively affected by heat stress in summer and are responsive to active cooling (Dado-Senn et al., 2020). One of the first studies to implement heat abatement via active ventilation with fans in preweaning Holstein calves reported that calves with access to daytime ventilation had greater average daily gain, feed efficiency, and lower respiration rates than their counterparts without access to daytime fans (Hill et al., 2011). Grouped-housed calves exposed to subtropical summer heat stress had elevated rectal temperatures, skin temperatures, and respiration rates, and decreased feed intake compared with calves provided airspeeds of 2 m/s at the calf level via the provision of fans (Dado-Senn et al., 2020). Female calves exposed to continuous ventilation via redirection of air inside outdoor hutches in summer had decreased skin temperatures and respiration rates when compared with heifers exposed to elevated temperature-humidity index in a continental summer and housed in outdoor hutches with passive ventilation alone (Dado-Senn et al., 2024). Although current efforts to develop effective heat abatement strategies for preweaning dairy calves are being investigated, much remains to be understood regarding potential immunomodulatory effects on the calf and its effects on morbidity and mortality. Herein, we evaluated immune and inflammation markers, including neutrophil (**NEU**) counts, NEU phagocytosis, and blood leukocyte gene expression, in outdoor hutch-housed dairy heifers with or without access to active ventilation during a continental summer. We hypothesized that providing continuous active ventilation to neonatal calves during the first 4 wk of life will improve immune status relative to heifers with passive (natural) ventilation, partially through improving NEU phagocytosis capacity and reducing inflammation markers.

All procedures were approved by the University of Wisconsin–Madison Institutional Animal Care and Use Committee (study #A006455). Thirty-two heifer calves were housed in outdoor individual sand-bedded polyethylene calf hutches (Calf-Tel, L. T. Hampel Corp.) at the University of Wisconsin–Madison Arlington Agricultural Research Station. At birth, all heifers were fed 3.8 L of colostrum (average Brix = 23.7%) within 4 h and were subsequently offered 2 quarts (1.89 L) of milk in 2 meals for the first 2 d. At 3 d of age, heifers were randomly assigned to hutches with passive (**PASS**; 0.07 m/s air speed inside the hutch, n = 32) or active ventilation (**ACT**; 1.1 m/s air speed via solar-powered fans, n = 32) until 28 d of age. The solar-powered ventilation system described by Dado-Senn et al. (2024) was slightly modified such that the 0.6-m ventilation tube was replaced with a bifurcated tube redirecting the air of one fan into 2 hutches. At d 28, the fans for the ACT group were turned off, and all heifers were under PASS until weaning (56 d of age). Details on the experimental design and ventilation system can be found in Larsen et al. (2025).

Blood samples were collected via jugular venipuncture at postnatal d 1 (24 h of age), 7, 14, 21, 28, 42, and 56 into 10-mL sodium heparin anticoagulant (BD catalog #367880, Franklin Lakes, NJ). Blood was set on ice before centrifugation at 3,000 × *g* for 20 min at 4°C to harvest plasma. Plasma fractions were aliquoted and stored at −20°C until analysis. Whole blood (3 mL) for the analysis of circulating leukocyte mRNA was drawn on postnatal d 28 into Tempus Blood RNA Tubes (#4342792, Thermo Fischer) containing 6 mL of Stabilizing Reagent. Following blood collection, the tubes were shaken vigorously for 30 s, placed on ice during transport, and frozen at −20°C until RNA was extracted. Two milliliters of blood was collected on d 1, 7, 14, 21, 28, 42, and 56 using K_2_EDTA anticoagulant (BD catalog #367856, Franklin Lakes, NJ) for complete blood count analyses. Blood was also collected on d 14, 28, and 42 using lithium heparin anticoagulant (BD catalog #367880, Franklin Lakes, NJ) for flow cytometry analyses.

A VetScan HM5 Hematology Analyzer was used to analyze whole blood on d 1, 7, 14, 21, 28, 42, and 56 (n = 16 per treatment). Blood samples were measured using the Zoetis Vetscan HM5 and reagent pack (#NC1831220, Thermo Fischer) within 2 h of collection for hematocrit percentage, RBC, and white blood cell (**WBC**) counts and WBC differential (counts and %), including hematocrit %, NEU, LYM, monocytes (**MON**), basophils, and eosinophils. Total plasma IgG concentrations (n = 12–16 per treatment) on d 1 (24 h after birth), 14, 28, 42, and 56 were quantified by radial immunodiffusion assay (Radial Immunodiffusion Test for Bovine IgG, Triple J Farms, #728411) according to the manufacturer's instructions with minor modifications. Briefly, samples were diluted in 0.9% saline (plasma 1:2) to fall within the standard curve range of the assay. Plasma glucose concentrations were quantified on the same days utilizing a colorimetric assay (Wako Autokit Glucose, Fujifilm #997-03001) according to the manufacturer's instructions.

Total RNA was extracted (n = 12 per treatment) from whole blood using the Tempus Spin RNA Isolation Kit (# 4342792, Thermo Fisher Scientific, USA). Following mRNA extraction, the quality and concentration of the extracted mRNA were analyzed using the Nanophotometer (#ND50-GO, IMPLEN-GO, USA). Extracted RNA was reverse transcribed to cDNA with iScript Reverse Transcription Supermix (#1708840, Bio-Rad, USA) and UltraPure DNase/RNase-free distilled water (#10977015, Thermo Fisher Scientific, USA) using a T100 thermal cycler (#1861096, Bio-Rad, USA, 5 min at 25°C, 46°C for 20 min, 1-min cycle at 5°C). The subsequent cDNA samples were diluted (1:3) in UltraPure DNase/RNase-free distilled water. Quantitative real-time PCR was conducted using the CFX96 Touch Real-Time PCR Detection system (#1854095 Bio-Rad, USA) using SsoFast EvaGreen Supermix (#1725204, Bio-Rad) per the manufacturer's recommendations. All samples were run in duplicate and all plates included duplicate positive (cDNA pool) and negative controls (UltraPure distilled water). The PCR cycling conditions were as follows: an initial denaturation at 95°C for 3 min, followed by 40 cycles of denaturation at 95°C for 10 s and annealing/extension at 55°C for 30 s, and a melt curve analysis from 65°C to 95°C with 0.5°C increments held for 5 s. For analysis, the geometric mean of 2 housekeeping genes (*RSP9* and β-actin) was used to normalize gene expression of heat shock factor 1 (*HSF1*), heat shock proteins (*HSP72* and *HSP90*), interleukins (*IL-2*, *IL-4*, *IL-6*, *IL-10*, *IL-12*, *IL-13*, *IL-17*, and *IL1-β*), toll-like receptors 2 and 4 (*TLR2* and *TLR4*), tumor necrosis factor-α (*TNF-α*), nuclear factor kappa B subunit 1 (*NFKB1*), transforming growth factor β (*TGFβ1*), *IFN-γ*, L-selectin (*CD62L*), co-stimulatory molecule (*CD28*), and haptoglobin (*HP*). Supplemental Table S1 (see Notes; Tabor, 2026) displays the primer sequence for all genes. Housekeeping gene selection was based on a previous study using immune cells (Marrero et al., 2021) and their expression remained stable across experimental groups in the present study (*P* > 0.22). All primer pairs displayed melting curves with a single peak, indicative of a pure, single amplicon, confirming the specificity of the primers. All efficiencies (10^(−1/slope)^− 1 × 100) ranged between 95% and 109%. Last, primer binding specificity was tested in silico against the target genome to avoid off-target amplification.

For phagocytosis and oxidative burst analysis, whole blood (100 µL) was transferred from sodium heparin tubes to three 5-mL Falcon tubes for each sample, labeled dihydrorhodamine 123 (**DHR**), DHR + phorbol 12-myristate 13-acetate (**PMA**), and DHR + propidium iodine (**PI**) *Escherichia coli*, respectively. Tubes were placed on ice, and 10 µL of diluted DHR was added to each sample. All tubes were vortexed and incubated in the dark at 37°C for 15 min. Following the first incubation, 20 µL of PMA working solution was added to tube 2 (DHR + PMA), and *E. coli*(#EC11303, Sigma-Aldrich, USA) at 40:1 NEU ratio to tube 3 (DHR and PI). Samples were incubated in the dark for 15 min at 37°C, and then placed on ice to stop phagocytosis and oxidative burst activity. Lysing buffer 1× RBC (2 mL, #00-4333-57, Thermo Fisher Scientific, USA) was added to remove RBC, and subsequently incubated in the dark for 10 min at 37°C. Cells were washed with 1 mL of PBS, centrifuged at 200 × *g* for 5 min at 4°C, and the supernatant was discarded. The previous step was repeated with 2 mL of cold staining buffer, and cells were resuspended in 250 µL of staining buffer.

Phagocytic and oxidative burst activity of granulocytes was quantified and analyzed via flow cytometry (Accuri C6, BD Biosciences, San Jose, CA) based on forward and side scatter (FSC/SCC) parameters using FlowJo10 software (version 10.10, TreeStar, Palo Alto, CA). Granulocytes were gated based on forward and side scatter (FSC/SCC) parameters. Neutrophils (representing greater than 90% of the granulocyte population) were identified on the basis of their size and complexity in the density cytogram, which were generated by linear amplification of the signals in the forward and side scatter channels. Data acquisition was completed when 10,000 events were acquired. The number of NEU in the control sample (DHR) was compared with the number of NEU in the positive control sample for both *E. coli*-stained PI and PMA. Upward cell migration indicates activation of NEU in response to chemotactic factors, and cell migration can be determined based on the change in forward scatter. Parameters quantified from the cytograms included the percentage of NEU that contained red fluorescence, indicating phagocytosis of PI-labeled *E. coli* (**PHA**), and the percentage of NEU with red and green fluorescence, indicating oxidative burst (**OB**; Martinez et al., 2012; Wójcik et al., 2020).

Data were analyzed in SAS using the MIXED procedure (v. 9.4, SAS Institute Inc., Cary, NC). Mixed models were used to analyze repeated measures variables, such as plasma IgG, glucose, and complete blood count. The model included treatment (ACT vs. PASS), time (days of age), and their interactions as fixed effects. Animal (ID) within treatment was used as a random effect. Measures on d 1 (before initiation of treatment) served as a covariate in the model, and were analyzed individually to ensure they were not different between groups and that randomization worked as expected. The PHA and OB were run with the same model for each individual day (14, 28, 42) due to 3 nonmatching animals across all time points. Normalized gene expression (ΔCt) on d 28 was analyzed using the same model without repeated measures. Data are displayed as the model estimates (ΔΔCt = ΔCt_PASS_ − ΔCt_ACT_). All residuals were tested for normality and homogeneity of variance. Variables were transformed to achieve normality when necessary. The LSM ± SE are presented unless otherwise noted. *P* ≤ 0.05 were considered statistically significant, and 0.05 < *P* ≤ 0.1 were considered tendencies.

Consistent with randomization, all d 1 baseline values did not differ between PASS and ACT groups (*P* > 0.22). Complete blood counts, glucose, and IgG were analyzed during the ventilation treatment period (d 7, 14, and 28, and on d 42 and 56 post-treatment).

During the ventilation treatment period, there was a treatment effect for the number of WBC, whereby PASS heifers had higher WBC, LYM, NEU, and MON counts relative to ACT heifers (*P* < 0.03, Figure 1). Total plasma IgG did not differ between treatments (22.8 vs. 18.4 ± 2.18 g/L; ACT vs. PASS; *P* = 0.29). Although glucose concentrations (98.6 vs. 97.5 ± 2.99 mg/dL; ACT vs. PASS) did not differ between treatments (*P* = 0.17), there was a treatment by day interaction (*P* = 0.05), whereby PASS heifers had higher circulating glucose at d 14 and 21, relative to ACT (114.1 vs. 90.8 and 106.6 vs. 93.2 ± 5.9 mg/dL, respectively). Calves with access to ACT ventilation had or tended to have greater percentage of NEU with phagocytic activity on d 14 (64.2 vs. 37.5 ± 9.74%, *P* = 0.07), d 28 (70.1 vs. 41.9 ± 8.6%, *P* = 0.03), and d 42 (59.4 vs. 21.5 ± 7.2%; *P* = 0.01; Figure 2). No differences were observed between groups during the ventilation period on d 14 and 28 for the percentage of NEU with OB (*P* > 0.52, Figure 2).

**Figure 1:**
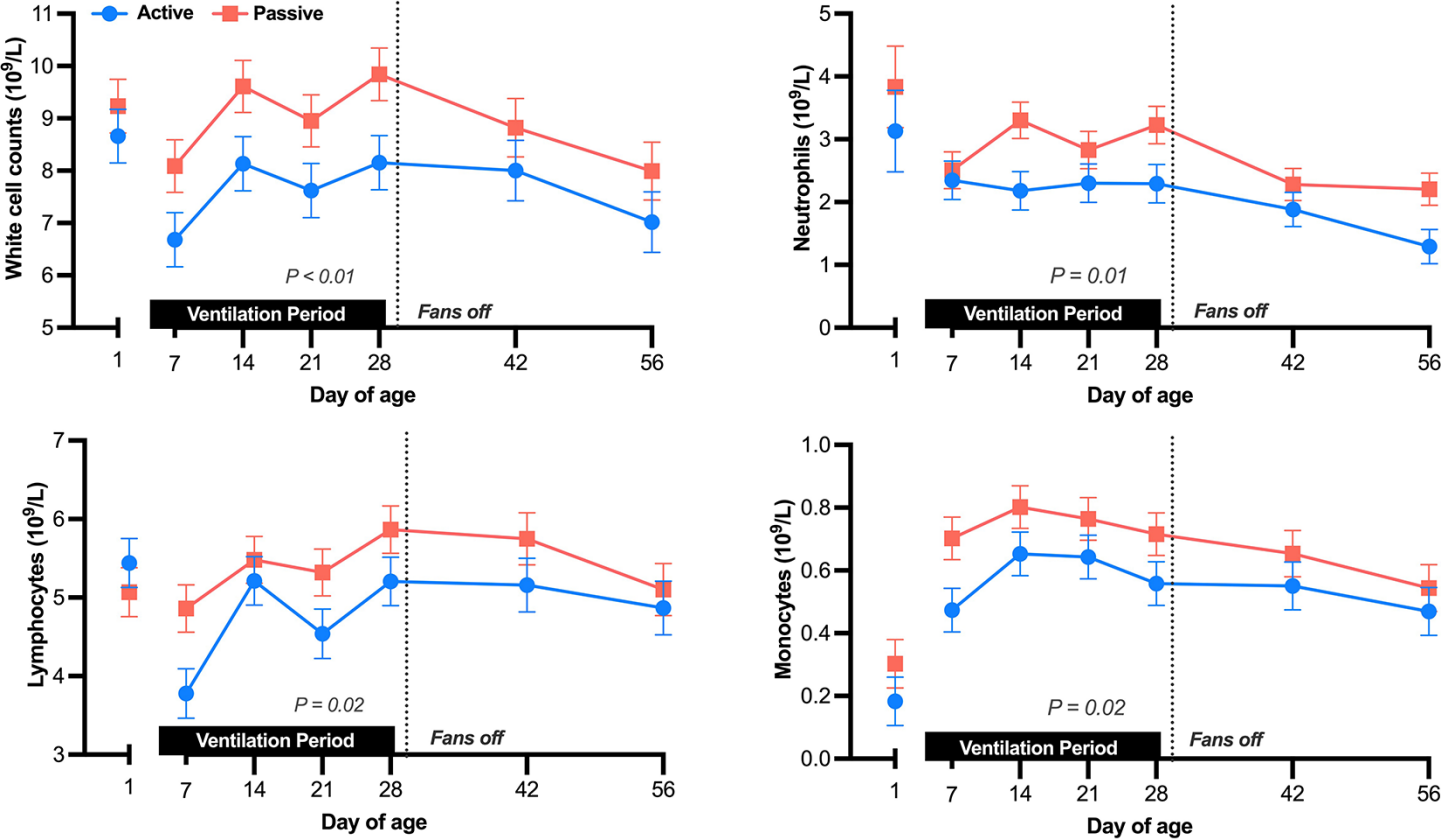
Circulating white blood cell, lymphocyte, neutrophil, and monocyte counts at d 1 of age (baseline) and during the preweaning period (d 7, 14, 21, 28, 42, and 56 of age) in Holstein heifers housed in outdoor hutches with active ventilation (solar-powered fans, blue lines and circles; n = 16) or passive ventilation (no fans, red lines and squares; n = 16) during summer. The black rectangle along the x-axis indicates the ventilation (treatment) period (d 3–28 of age); fans were off during the postventilation period (d 29–56). *P*-values (main effect of treatment; no interactions) are shown for each period. Data presented as LSM ± SEM.

**Figure 2:**
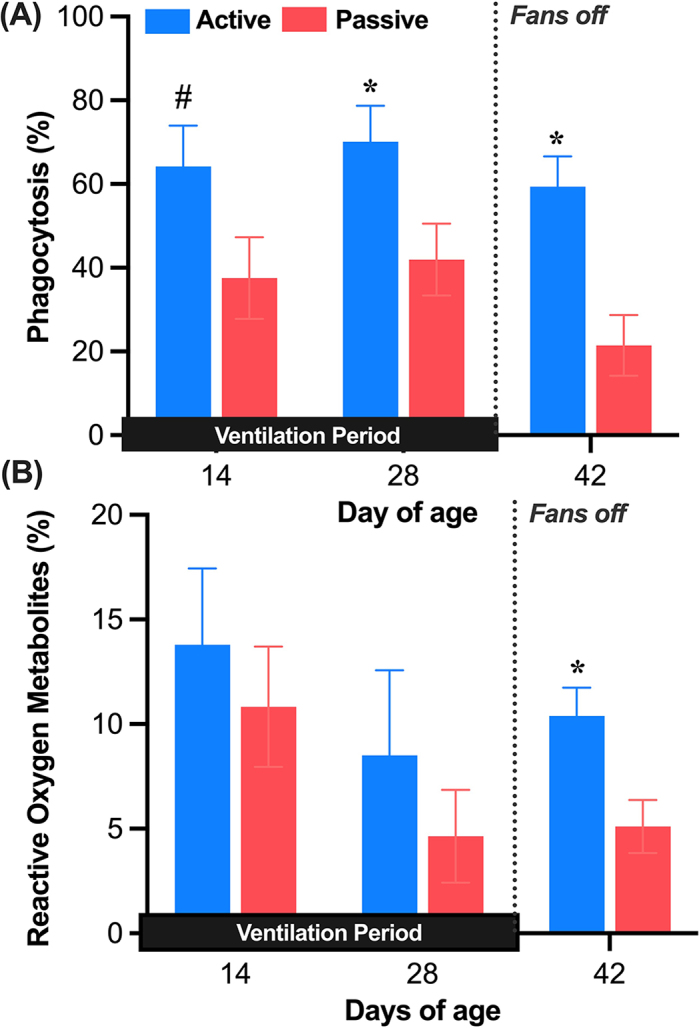
Percentage of granulocytes with phagocytic activity (A) and associated reactive oxygen metabolite production (B) in blood samples collected on d 14, 28, and 42 from Holstein heifers housed in outdoor hutches with active ventilation (blue bars, 1.1 m/s; n = 12) or passive ventilation (red bars, 0.05 m/s; n = 12) during summer. The black rectangle along the x-axis denotes the ventilation (treatment) period (d 3–28 of age); fans were off during the postventilation period (d 29–56). *P* ≤ 0.01 (*) indicates significance differences between treatments, and 0.05 < *P* ≤ 0.1 (#) indicates a tendency. Data presented as LSM ± SEM.

When analyzed during the postventilation treatment period (d 42–56), PASS heifers continued to have greater NEU counts relative to their ACT counterparts (*P* = 0.01, Figure 1). Additionally, glucose concentrations were greater in ACT relative to PASS heifers (*P* = 0.02). The percentage of NEU with OB was greater in ACT relative to PASS heifers at d 42 (10.4 vs. 5.11% ± 1.3%, *P* < 0.01, Figure 2). The mRNA expression of HP, IFN-γ, and NFKB1 was downregulated (all, *P* < 0.04) in peripheral leukocytes of ACT relative to that of PASS heifers (Figure 3). The mRNA expression of IL-12, -6, and -4 was also downregulated (all, *P* < 0.04), and IL-8 tended to be downregulated (*P* = 0.09; Figure 3) in peripheral leukocytes of ACT relative to that of PASS heifers. The mRNA expression of CD28 and HSP72 was upregulated, and TGF-β1 tended to be upregulated (all, *P* < 0.06; Figure 3) in peripheral leukocytes of ACT heifers. No differences in mRNA expression were found between ACT and PASS peripheral leukocytes for all other genes (*P* > 0.10; Figure 3).

**Figure 3:**
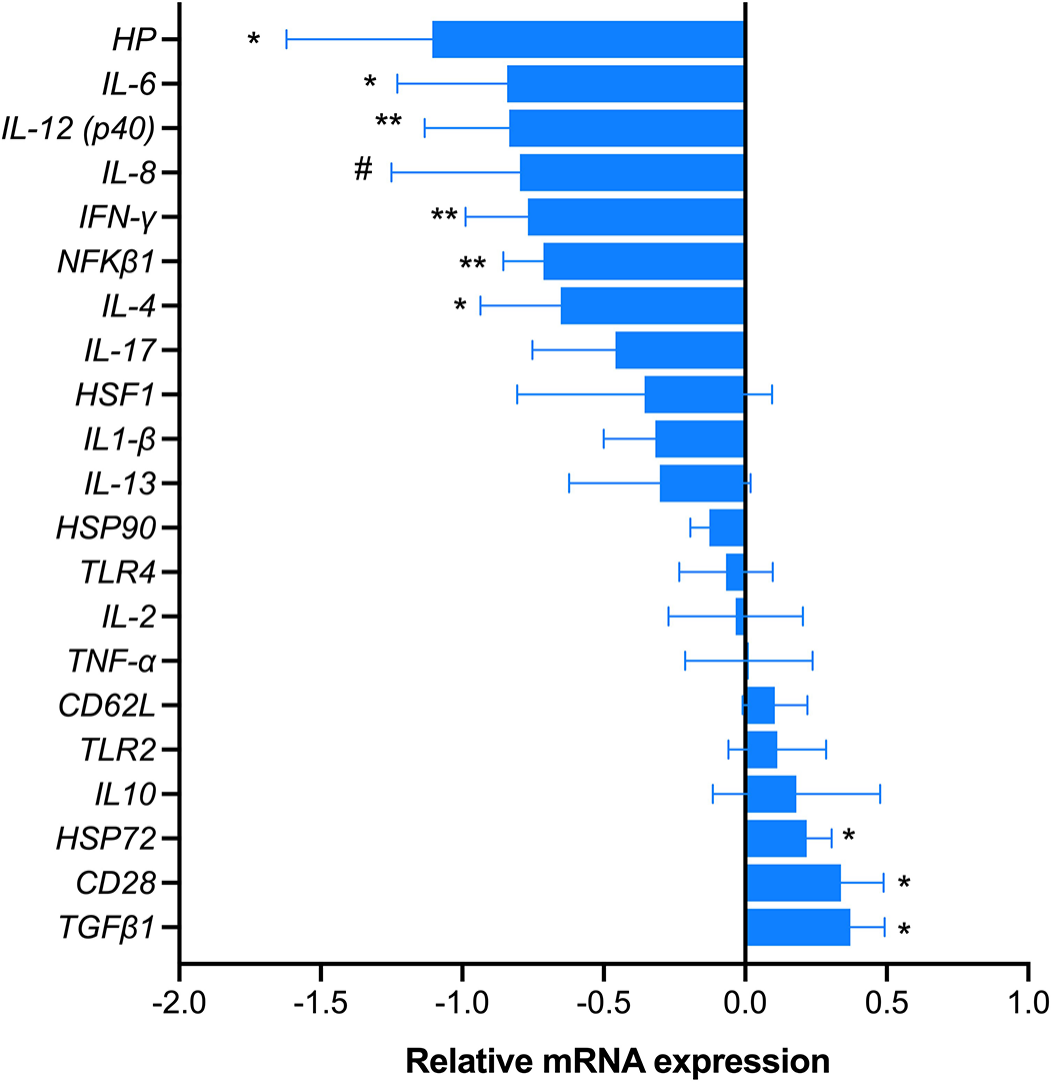
Peripheral blood leukocyte mRNA expression from 28-d-old Holstein heifers housed in individual outdoor hutches with active ventilation (ACT, 1.1 m/s; n = 12) or passive ventilation (PASS, 0.05 m/s; n = 12) during summer. Expression of target genes associated with immune and inflammatory responses was quantified. The geometric mean of *RSP9* and β-actin housekeeping genes was used for normalization (ΔCt calculation). Housekeeping genes had stable expression across experimental groups (*P* > 0.22). Data are presented as the model estimates (relative mRNA expression ΔΔCt = ΔCt_PASS_ − ΔCt_ACT_) ± SEM. Negative and positive ΔΔCt indicated downregulation and upregulation in ACT relative to PASS, respectively. *P* ≤ 0.01 (**) and 0.01 < *P* ≤ 0.05 (*) indicate significance, and 0.05 < *P* ≤ 0.1 (#) indicates a tendency.

In the current study, we tested an improved version of our original prototype for active ventilation via solar-powered fans (Dado-Senn et al., 2024) to provide heat abatement to preweaning dairy heifers. This system achieved 1.74 m/s air speed at calf height level inside outdoor hutches directly exposed to the elements. Heifers with access to hutch active ventilation in summer had lower total WBC, LYM, and NEU counts, increased neutrophilic phagocytic and OB activity, and lower expression of pro-inflammatory genes compared with calves under the same environment but with naturally ventilated hutches.

Heat stress–induced changes in immune function have been documented in cattle, with alterations to cell-mediated immunity having a significant impact on preweaning calf immunocompetence, which may render an animal more susceptible to infection (Dahl et al., 2020). Neutrophils, a primary phagocytic leukocyte, increase in circulation in response to stress, inflammation, or infection, whereas LYM modulate immune defense (Davis et al., 2008). In this study, calves under active ventilation had decreased circulating NEU compared with their heat-stressed counterparts, which could be indicative of decreased immune stress response. It has also been reported that transport stress led to an increase in total leukocyte and NEU numbers in lactating dairy cows (Hong et al., 2019). In the current study, calves exposed to summer heat stress via passive ventilation tended to have increased WBC, NEU, and LYM counts compared with their actively ventilated counterparts. The higher NEU counts under heat stress are consistent with previous stress findings (Yagi et al., 2004; Hong et al., 2019).

Interleukin-8 is a cytokine that primarily affects NEU function, and it is produced in response to pro-inflammatory cytokines (e.g., TNF-α, IL-1β). Interleukin-8 causes NEU to migrate toward the site of infection and stimulates phagocytosis once present. A previous study found a significant upregulation of IL-8 gene expression, as well as the *CXCR1* gene, which codes for an IL-8 receptor on NEU, after intramammary infusions of live lactococci (Crispie et al., 2008). This supports our findings, as IL-8 mRNA expression and circulating NEU were both higher in heifers housed in hutches with passive ventilation compared with those housed in actively ventilated hutches in summer, suggesting an inflammatory or infectious state in these animals. In the early stages of inflammation, cytokines such as IL-6 are produced by T cells, which activate inflammatory pathways. The NF-κβ complex functions as a transcription factor to regulate multiple biological processes, including immune function and inflammation (Ran et al., 2004). This pathway is also required for the induction of inflammatory genes, such as IL-6 and IFN-γ, and granulocyte macrophage-colony stimulating factor (GM-CSF; Yu et al., 2022). Previous studies have shown heat stress negatively affects this pathway, resulting in increased inflammation and overexpression of inflammatory genes (Martínez et al., 2021). These findings are in concordance with the downregulation of pro-inflammatory cytokines IL-6, IL-8, and IFN-γ observed in the actively ventilated heifers, as well as the decreased number of circulating NEU.

The preweaning period is a period of developmental plasticity for the immune system of dairy calves and is generally associated with increased rates of respiratory infections (Hulbert and Moisá, 2016). Neutrophils are essential elements of the innate immune system, which ensure defense through the processes such as phagocytosis and OB (Gierlikowska et al., 2021). It is well known that heat stress exacerbates illnesses during early-life window of susceptibility (Dahl et al., 2020) due in part to the detrimental effects on NEU functionality (Marrero et al., 2021). An in vitro assessment of the effects of heat stress on phagocytosis and OB in bovine PMN cells found that the phagocytosis and OB rate was reduced when PMN cells were incubated at 41°C relative to cells incubated at 39°C (Lecchi et al., 2016). Heat-stressed periparturient mature Holstein cows have decreased NEU reactive oxygen species and phagocytosis (do Amaral et al., 2011). Herein, the percentage of NEU with phagocytic activity was greater on d 28 and 42 in the active compared with the passively ventilated heifers. Along with this, a greater percentage of NEU with OB was observed on d 42 in actively ventilated heifers. In accordance with previous literature in cattle, these findings suggest that phagocytosis and OB activity in NEU that present in calves exposed to heat stress in summer are severely impaired and that provision of active ventilation may be beneficial in improving NEU function.

This study offers insights into the immunomodulatory advantages of a new heat abatement management strategy for preweaning dairy calves. The provision of continuous active ventilation via solar-powered fans to outdoor hutches during a continental summer generated air speeds at calf height that enhanced conductive cooling lowering thermal indices (Larsen et al., 2026) and attenuated immune-inflammatory cell responses in growing preweaning heifers. A limitation of the present study that further research needs to address is the lack of characterization of the developmental trajectory of specific cell populations under these conditions, and the long-term effects on health and survival of heifers with access to continuous active ventilation in hutches.
